# A Review, and a Forecast

**Published:** 1920-11-06

**Authors:** 


### THE HEALTH OF THE ENGLISH PEOPLE.

### A Review, and a Forecast.

Last week mention was made in these pages of one section, namely, maternity and child welfare, in that remarkably comprehensive review which recently came to hand as the First Annual Report of the Health Ministry's Chief Medical Officer. There are few departments of general health work that Sir George Newman's analysis does not include, and it is, perhaps, not surprising that the majority of public comments appearing so far have failed to summarise more than a very small part of this somewhat remarkable publication. The Chief Medical Officer and his colleagues have given us a volume in which they undoubtedly do their utmost to put all the scientific, if not the political, cards on the table, and it is correspondingly difficult simultaneously to comprehend them all. One critic remarks that the whole is largely a repetition of what has been repeatedly said before. Applied to some sections this remark is perhaps justifiable. But, nevertheless, matters of such urgent public importance are dealt with that the crime of repetition may, at the present juncture, well be forgiven.

In an introductory note Sir George Newman outlines the purpose and principal functions of the Ministry. Here it is unnecessary to detail his explanations, as practically his own summary thereof was published in our report of one of his most recent speeches (The Hospital, October 23); but the fact which he would emphasise is that the public must not stand quietly by and expect too much from the mere establishment of a centralised health department. It cannot automatically solve the many problems of local autonomy and administration, and it must for the moment signify a first step in a great national scheme of unification in health work.

"It must not be forgotten that the Ministry, as such, is but a part of an organised scheme for the national health. An organised scheme in any country must include (a) a central supervising department; (b) local authorities or representative bodies for the actual carrying out of the policy of such a Ministry in the local divisions or areas; (c) voluntary societies and agencies; (d) *educated people, willing and able to practise the way of health.* These are four factors, but the foundation of their operations, as well as their direction, must be the advance of our knowledge of the science and art of medicine. Hence there is a fifth factor, the medical practitioner—in some ways the most important and essential factor of all. Upon him the State has been steadily imposing, for many years, specific duties in regard to the communal health, though it has been slow to associate him closely with its operations. *One of the principal objects of the Ministry of Health is to secure his co-operation* as an integral and active force in the physical well-being of the nation."

The italics in this quotation are our own, and they emphasise two of the most vital points in the Ministry's objective. Concerning the first, we have repeatedly urged and applauded every new departure in the health education of the general public. Therein probably lies the cure for many ills other than those of a directly hygienic bearing. But concerning the second there has been no little doubt prevalent, and although the Report does not enter into the argument of State *versus* voluntary medical service, yet the author remarks that henceforward the general practitioner will become an integral part of the national organisation of Preventive medicine. This will not, as suggested by some critics, "abolish" him, nor yet will it absorb him into a whole-time national service. It is the Ministry's stated opinion that neither of these courses is either practicable or desirable. Therefore we shall apparently not as yet see anything resembling a general State medical service, and the Ministry's scheme of gradual education for both doctor and patient will be first given a thorough trial. This plan will undoubtedly receive the approval of the great majority of English medical men, and there seems little doubt that it is the most satisfactory medico-political attitude that officialdom could adopt at the present time.

Sir George Newman is at great pains to sketch out the arrangements and machinery by which the standard of insurance practice is to be improved. Some measures are already introduced. There are increased remuneration, improved record cards, the whole-time regional medical officers, and the actual limitation in size of individual "panels." Around each of these have arisen, and round some still arise, somewhat thorny problems, and they have provided a truly happy hunting ground for the critics, but one is glad to see them, because move we must, and the sooner the Ministry's present schemes stand or fall by their own proven value, the sooner will ultimate improvement come in the actual health of the people. At least a free and needed revision of panel practice is actually taking place, and meanwhile Sir George Newman, recognising that much still remains to be done, appears to keep an open mind and a close watch on the individual measures as they are severally introduced.

### The Physical Survey.

The question of hospital service in this country is not directly dealt with in the report, but the Ministerial attitude is already referred to on an earlier page (p. 111). Thus the next section in order of general interest is probably that relating to the actual physical survey of the people which Sir George Newman has made from data already collected. It is both obvious and highly satisfactory that here and there considerable improvement has been made in the health of the nation, and that the death-rate is declining. Meanwhile, it must be realised that a highly serious amount of preventable sickness and avoidable disablement remains. It is unnecessary again to outline the indirect influence

**The Health of the English People**—(*continued*).

of such conditions on the physical stamina of a nation, for the whole crystallises into the fact that above all we must aim to prevent rather than cure physical impairment. In the first place we have to rear and maintain a healthy race of people, and meanwhile to continue our attack upon infection and actual disease. It is impossible to improve on the author's summary of the present situation, which, in brief, runs as follows:

1. There is a steadily falling birth-rate, which in 1919 reached the figure of 18.5.

2. There is a death-rate of 13.8, which shows a steady decline at all ages, thus raising the expectation of life from birth upwards.

3. The infant mortality rate (89 per 1,000 births) is one of the lowest recorded, yet we must realise that there is still much unnecessary loss of life, both of mother and child.

4. There are over half a million cases per annum of infective or epidemic disease, the ultimate results of which are graver than the primary burden.

5. Tuberculosis, measles, acute rheumatism, and influenza are still relatively prevalent, and, with venereal disease, are among the great causes of physical disablement.

6. There is a mass of disability and incompetency from relatively trivial sickness, which in its early stages is relatively neglected.

7. The systematic medical inspection of school children has revealed a substantial degree of physical and mental impairment, both the existence and effect of which have been abundantly confirmed by the examination of recruits.

8. The expenditure on sickness and disablement benefit and other similar returns denote a very serious amount of time lost from unemployment, amounting to no less than 260,000 years per annum for the insured population only.

It is perfectly true that these facts and figures have individually been brought to the public notice before, but in his present report Sir George Newman has portrayed in a singularly valuable way the real health situation in this country. The work of necessary organisations, the League of Nations, the League of Red Cross Societies, and others, is all described in its true bearing upon the Ministry's activities. The valuable improvements in the sanitary control of infectious disease, the small-pox and vaccination problems, the revision of Port sanitary requirements, and a host of other changes, large and small, are outlined and to a great extent explained. Whatever the public has expected from the Ministry, whether it is disappointed in its expectations or not, Sir George Newman's report shows that the year covered has been replete with activity, the great bulk of which has undoubtedly been wisely directed.

